# The impact of psychosocial safety climate on public sector job satisfaction: the moderating role of organizational climate

**DOI:** 10.1186/s40359-023-01513-8

**Published:** 2024-01-19

**Authors:** Albert Joseph bin James Lintanga, Balan Rathakrishnan

**Affiliations:** Fakulti Psikologi dan Pendidikan, Universiti Malaysia Sabah, Sabah, Malaysia

**Keywords:** Job satisfaction, Organizational climate, Psychological safety climate, Public sector

## Abstract

**Background:**

The purpose of this study is to uncover the effect of psychological safety climate (PSC) on employees’ job satisfaction and organisational climate mediating processes explaining that association. It is posited that the four PSC aspects (management commitment, management priority, organisational participation, and organisational communication) are important for employees’ job satisfaction and organisational climate act as resources to facilitate the enactment of managerial quality.

**Methods:**

This study uses a quantitative approach through a questionnaire survey method involving 340 Kota Kinabalu City Hall employees who were selected through simple random sampling.

**Results:**

The results of linear regression analysis found that organisation participation has a positive significant relationship with job satisfaction. Organisational communication also showed a negative and significant relationship with job satisfaction. Meanwhile, both management commitment and management priority are statistically insignificant. When the organisational climate is included in the relationship as a mediator through Structural Equation Modelling (SEM) to reinforce the role of psychological safety climate in increasing job satisfaction, such mediating role can only strengthen the relationship between management commitment and organisational participation with job satisfaction.

**Conclusion:**

Despite the study being cross-sectional, it contributes to knowledge on the resources facilitating PSC, which is important for employees’ psychological health. From a practical viewpoint, this study contributes to the literature showing that organizations with good PSC should have policies and practices directed towards employee well-being. The implications of the study for DBKK management are to providing knowledge on the types of psychosocial safety climate domains that plays a crucial role in improving the job satisfaction of DBKK employees.

## Introduction

In contemporary times, organizations are increasingly recognizing the significance of employees as valuable assets in delivering high-quality services and products to consumers. Employees with a high level of job satisfaction play a crucial role in ensuring the success of an organization. Such individuals exhibit better work results, heightened performance, reduced absenteeism, and a sustained loyalty to the workplace [1]. High job satisfaction also has the ability to influence a positive work environment and provide benefits to both the organization and the employees themselves. In general, organizations need to pay attention to their employees’ job satisfaction due to the numerous negative effects associated with low job satisfaction. These effects may manifest as demonstrations or strikes by dissatisfied employees, who are discontent with the rewards they receive, find their wages inadequate in relation to the tasks and responsibilities they undertake, or struggle to meet the needs and demands of the cost of living. This stands in contrast to employees with high job satisfaction, who positively impact the organization by contributing to increased efficiency and productivity [2].

The Psychosocial Safety Climate (PSC) is gaining increasing attention in the field of organizational behavior studies. PSC pertains to the perception of practices and procedures within an organization that should be communicated to its employees, emphasizing the importance of psychosocial health and employee safety at work [3, 4]. Psychosocial safety climate is a way to increase comfort and safety in doing work which has an important impact on individual job satisfaction [5]. The chairman of the National Institute of Occupational Safety and Health (NIOSH) stated that a positive psychosocial environment can benefit employees’ mental health, foster a sense of social inclusion, enhance identity and status, provide opportunities for development, and boost employee confidence. Conversely, a poor psychosocial work environment can have significant negative effects on employee health. Furthermore, the neglect of mental health and psychosocial factors in the workplace not only harms employees but also directly impacts the productivity, efficiency, and output of the organization.

However, research on this topic is predominantly conducted in European countries, Australia, and the United States. Only a handful of researchers have examined the importance of the psychological safety climate aspect in Asia, particularly in Malaysia. Despite the crucial relationship between psychological safety climate and job satisfaction, research findings suggest that discussions on the role of psychological safety climate remain limited in the context of work culture in Malaysia. Emphasizing the psychological aspect and employee well-being influenced by the psychological safety climate is integral to understanding the elements contributing to job satisfaction [6]. The psychological safety climate has been identified as a crucial factor capable of alleviating emotional fatigue among employees. It has been asserted that the implementation of a psychological safety climate, focusing on well-being and psychological health, can effectively reduce the stress associated with workloads and mitigate the potential for other mental health disorders [6]. Building upon the researcher’s preliminary investigation into previous studies in this decade, it is evident that there is a scarcity of research that delves into the dimensions of the psychological safety climate. Specifically, studies considering aspects such as management commitment, management priorities, organizational involvement, and organizational communication in relation to job satisfaction are notably limited.

In the meantime, the organizational climate serves as a crucial instrument for achieving organizational goals and upholding the physical and psychological well-being of employees. Organizational climate, in essence, encapsulates employees’ perceptions of the surrounding work environment, encompassing their understanding of the organization’s policies, practices, rewards, and procedures. In contrast, a psychosocial safety climate is characterized by organizations prioritizing the psychological health and safety of employees. This entails organizations playing a pivotal role in cultivating a positive work environment, offering support to employees, and addressing issues that may lead to work-related stress. Organizations fostering a strong psychosocial safety climate are characterized by the implementation of policies and practices centered around enhancing employee well-being.

Considerable research has been conducted on organizational climate, psychological safety climate, and job satisfaction. However, most studies have focused on the independent relationships, such as between organizational climate and job satisfaction [7], psychological safety climate and job satisfaction [8], organizational climate and organizational commitment [9], organizational participation and job satisfaction [10]. Only a limited number of researchers have delved into the intricate connections between various aspects of organizational behavior and their practical applications within organizations. There is a noticeable gap in knowledge and awareness regarding the crucial role of organizational climate and psychological safety climate in influencing job satisfaction [11]. In contrast to developed countries where the link between psychological safety climate and organizational climate has led to the creation of specific regulations and laws on employee safety, developing countries like Malaysia still grapple with issues due to a lack of awareness about psychological health and safety among employees and employers [6, 11].

The global exchange of knowledge among cultures in Asia provides an opportunity to assess the effectiveness of current theories and offers guidance for management decision-making. Despite this potential, only a handful of researchers have focused on the Asian environment, and very few studies are specifically relevant to Malaysia [6, 11]. Therefore, this study aims to contribute to the existing knowledge base from a Malaysian perspective. In light of the above, the psychological safety climate, which emphasizes the psychological health and safety of employees, has prompted researchers to explore the relationship between its components (management commitment, management priorities, organizational involvement, and organizational communication) and job satisfaction. The study also seeks to examine the influence of organizational climate on job satisfaction and psychological safety climate. This research is particularly pertinent in the Malaysian context, given recent issues in the public sector, including fraud, governance failures, workplace accidents, low customer satisfaction, and a high turnover rate. These issues raise questions about public sector accountability and diminish public trust. Thus, the study aims to assist management in identifying factors that can enhance organizational accountability by implementing sound psychological safety climate practices and organizational climate interventions to boost job satisfaction.

## Literature review

### Theoretical framework

#### Psychological safety climate

The PSC theory, highlighted by Idris [6], is a work stress theory within the domains of workplace health and safety and organizational psychology. PSC, in this context, encompasses employees’ perceptions of the systems, policies, practices, and procedures aimed at safeguarding the psychological health and safety of employees within their organization [6, 11]. The theory becomes apparent when management places value on and prioritizes the psychological health and safety of employees by offering meaningful, manageable work with sufficient resources while minimizing psychological risks. Consequently, PSC is posited as a source of work stress influencing employee job satisfaction [12]. The PSC theory plays a pivotal role in addressing the question of “where do job demands and resources come from?” In the context of this study, psychological safety climate is defined as a secure work environment provided by the organization to its employees through the implementation of policies, practices, and procedures designed to safeguard both physical safety and psychological health. This definition aligns with the four domains of psychological safety climate as emphasized [6, 11].

### Management commitment

Management commitment refers to addressing issues related to the psychological health of employees promptly and efficiently identifying and resolving problems. As per Dollard and Bakker, management commitment involves taking swift actions to rectify issues impacting the psychosocial health of employees. These prompt actions demonstrate the commitment of management to supporting the welfare of its employees, prioritizing their well-being over dealing with psychosocial stress issues. Laschinger, Shamian and Thomson debates management commitment as employees’ perception of the organization they work for [13]. It underscores three aspects of organizational commitment: trust and acceptance of the organization’s goals and values, employees’ willingness to strive toward achieving the organization’s goals, and the desire to remain a member of the organization. Management commitment is the level of employees’ confidence in accepting the organization’s goals, influencing their loyalty to be part of the organization [14]. Highly committed employees tend to be more responsible in carrying out their tasks [15]. Numerous studies indicate that employees who are satisfied with their jobs are more committed to the organization [16].

### Management priority

Management priority occurs when an emphasis is placed on the psychological health and safety of employees over production goals. This refers to a managerial approach that prioritizes the psychosocial well-being of employees. Indicators of management priorities manifest in policies, procedures, and work practices that positively influence employees’ work without compromising their psychosocial health. The perception of consistency or inconsistency between managers’ words and actions, observed over time, plays a crucial role. In an organizational context, when employees perceive alignment between their managers’ words and actions, a sense of cohesion emerges [17]. Managers who genuinely prioritize the psychological health of employees demonstrate this through supportive actions [18]. When employees recognize alignment between policies and the prioritization of psychological health, safety signaling mechanisms are triggered. This fosters an environment where employees feel secure in utilizing support and resources to mitigate or prevent emotional exhaustion at work.

### Organizational participation

Organizational Participation entails the active involvement of employees at all levels in raising awareness and safeguarding the psychological health and safety of the workforce. It involves collaborative efforts between management and employees in the safety process, achieved through engagement in consultations. The overarching goal of this collaboration is to prevent work-related stress experienced at the organizational level [19]. The approach of management and employee participation is rooted in individual or group behavior within communication flow and is integral to the decision-making process [20]. This approach empowers both management and employees with the responsibility and accountability for joint decision-making, ensuring the active involvement of both parties in achieving the goals and objectives related to employee psychological safety.

### Organizational communication

Organizational Communication provides a secure and effective channel for employees to voice their complaints and concerns, ensuring they feel safe even in unsatisfactory conditions. This type of communication, practiced by management and employees, addresses issues that may impact the safety and psychosocial health of employees. The emphasis on good and effective communication is crucial for distributing information more efficiently within the organization [18]. When management prioritizes employees’ psychosocial safety through organizational communication, it mitigates the impact of job demands on employees’ emotions [21]. This proactive approach at the organizational and management levels positively influences the work environment, aligning work demands and resources with organizational goals. Psychological safety communication and feedback involve a two-way communication process for exchanging information and addressing issues related to psychological safety between management and employees, ultimately enhancing the overall safety of the work environment [20].

### Job satisfaction

The Two-Factor theory of job satisfaction is often used to explain work motivation and its impact on individual job satisfaction. According to this theory, two key aspects of job satisfaction are highlighted. First, individuals reflect on when their job is rewarding and why they feel that way. Second, they consider when their job feels meaningless and the reasons behind that sentiment. Job satisfaction, based on the work environment, can decrease if not managed well. This includes various factors such as policies, supervision, compensation, interpersonal relationships, and working conditions. On the other hand, motivation that promotes job satisfaction is achieved by fulfilling individual needs related to meaning and personal development. According to this theory, job satisfaction is explained based on these two factors, both of which require the organization’s responsibility in providing a work environment that safeguards the well-being of their employees.

Job satisfaction is defined as an individual’s attitude or feelings towards their job [22]. A positive attitude and enjoyment in one’s job indicate higher job satisfaction, while a negative attitude and dislike for one’s job reflect lower job satisfaction [23]. Additionally, job satisfaction encompasses an employee’s overall feelings or attitude towards various job components, including the work environment, job characteristics, rewards, and communication with colleagues [24]. It can be viewed as a series of pleasant or unpleasant feelings and emotions that an employee experiences in relation to their job [25]. Scholars have noted that job satisfaction signifies positive attitudes and feelings that employees harbor towards their work [26]. Job satisfaction is a complex concept, dependent on various factors, and it varies from one employee to another. In the context of this study, job satisfaction is defined as the positive or negative attitudes and emotions that employees hold towards their jobs.

### Organizational climate

McClelland’s Three Needs Theory identifies achievement, affiliation, and power as key motivators, asserting that these needs are influenced by the organizational climate [27]. Organizational climate is a concept describing the subjective quality of the organizational environment, shaped by the perceptions and experiences of its members [27]. This concept comprises six necessary dimensions: (1) structure, where employees are well-organized with clear roles and responsibilities; (2) standards, measuring the pressure felt to improve performance and employee dignity; (3) responsibility, reflecting employees’ sense of self-leadership; (4) recognition, the feeling of being appropriately rewarded for job completion; (5) support, encompassing trust and mutual support within work groups; and (6) commitment, the sense of pride and commitment as a member of the organization [27]. Organizational climate is further defined as the functionality of employees and their interactions within the organizational environment [28]. It is described as the atmosphere of the internal environment based on how members perceive activities geared towards achieving organizational goals [29]. Alternatively, organizational climate is viewed as an overview of the experiences of employees in an organization [30]. This perspective is behavior-oriented, supported by research indicating that organizational climate influences productivity through emotional, learning, and behavioral changes in employees [31].

Next, it is stated that climate refers to the contextual situation at a point in time and is associated with the feelings and behavior of members of the organization [32]. Therefore, it is limited, subjective, changeable, and can be influenced and manipulated by the power dynamics of individuals. The study further indicates that organizational climate and organizational culture impact the social context within the organization [32]. Assessing organizational climate serves as a barometer for gauging individual opinions and perceptions towards the organization and its leadership, which may, in turn, influence their performance. In the context of this study, organizational climate is defined as the working environment practiced by an organization that significantly influences the methods of working and the behavior of employees within the organization. This working environment encompasses the subjective, changeable, and contextual aspects that can be shaped and influenced by individuals and power dynamics.

### Hypotheses development

#### The effect of psychosocial safety climate on job satisfaction

In a study investigating the connection between human resource management practices, organizational commitment, and job satisfaction, it was found that organizational commitment exhibits a positive and significant relationship with job satisfaction [33]. Other studies emphasize the importance of organizational commitment, asserting that it plays a crucial role in influencing the level of employee turnover [34]. The research suggests a positive correlation between organizational commitment and job satisfaction.

*H*_*1.1*_ *: There is a significant relationship between management commitment and job satisfaction of DBKK employees.*

In a study conducted by [35], aiming to investigate the influence of organizational priorities, job satisfaction, and organizational commitment through public accountability on organizational performance, it was found that there is a significant relationship between organizational priorities and job satisfaction. Furthermore, their study stated that the success or failure of a company is mostly determined by organizational and leadership priorities. They concluded that there is a positive effect of management priority and leadership style on job satisfaction [36].

*H*_*1.2*_ *: There is a significant relationship between management priority and job satisfaction of DBKK employees.*

In his study on the impact of employee participation on job satisfaction, Chapagai found that there is a significant relationship between employee participation and job satisfaction [37]. The findings of the study highlight that employee participation is a crucial factor in determining job satisfaction. Tourani and Rast expressed the same view and suggested that organizations should emphasize the involvement of employees and managers because it can improve their efficiency and performance [38].

*H*_*1.3*_ *: There is a significant relationship between organizational participation and job satisfaction of DBKK employees.*

In their study on the effectiveness of organizational communication on job satisfaction, Darijani, Soltani and Pourroostaei found a significant relationship [39]. Another study conducted by Erogluer on the relationship between organizational communication and factors affecting job satisfaction also found that there is a significant relationship between organizational communication and the level of employee job satisfaction [40].

*H*_*1.4*_ *: There is a significant relationship between organizational communication and job satisfaction of DBKK employees.*

### The moderating role of organizational climate on the association between psychosocial safety climate and job satisfaction

In their study on the influence of transformational leadership style and organizational climate on organizational commitment, Widyastuti and Manara found that there is a correlation between transformational leadership style and organizational climate on organizational commitment [41]. This opinion is also supported by Suarningsih, Alamsyah and Thoyib in their study to identify the relationship between organizational climate, organizational commitment, and employee performance. They found that organizational climate affects work performance indirectly through organizational commitment [42].

*H*_*2.1*_ *: Organizational climate moderate the relationship between management commitment and the job satisfaction of DBKK employees.*

Researchers studied the influence of organizational climate in the relationship between organizational priorities and work performance on 187 employees in the Malaysian broadcasting industry [39]. They found a significant relationship between organizational priority and organizational climate on work performance, confirming that organizational climate acts as a moderating variable for high-performance work systems in organizations. In their study analyzing the influence of organizational climate and organizational priorities on employee performance, they also found that organizational climate and organizational priorities simultaneously influence employee work performance [43].

*H*_*2.2*_ *: Organizational climate moderate the relationship between management priority and the job satisfaction of DBKK employees.*

In their study on the influence of leadership, commitment, and employee participation, with organizational climate as a moderator in the relationship between variables, researchers found that leadership influence is a significant predictor of organizational climate, commitment, and employee participation [44]. Additionally, the study on mediating effects found that organizational climate serves as a significant moderator between the influence of leadership and employee participation.

*H*_*2.3*_ *: Organizational climate moderate the relationship between organizational participation and the job satisfaction of DBKK employees.*

In their study examining the influence of organizational communication, organizational climate, and leadership on employee performance, researchers found that organizational communication, organizational climate, and transformational leadership significantly affect employee performance [45]. This opinion was reiterated in another study focusing on the influence of organizational climate on employee productivity [46].

*H*_*2.4*_ *: Organizational climate moderate the relationship between organizational communication and the job satisfaction of DBKK employees.*

### Conceptual framework

**Fig. 1:**
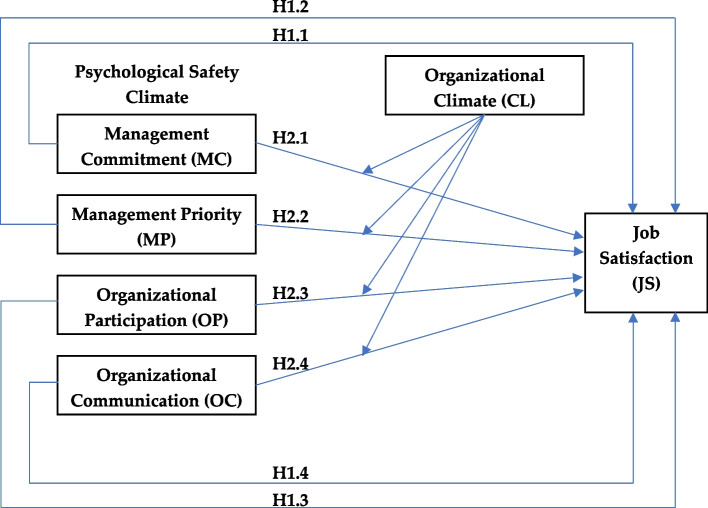
Proposed model

Building on the hypotheses development, this study investigates the effect of PSC on job satisfaction, with the mediating processes of organizational climate elucidating the underlying associations (refer to Fig. 1).

### Research design and methodology

Drawing inspiration from the Two-Factor Theory of job satisfaction and the Psychosocial Safety Climate (PSC) Theory, as highlighted by [27]. this study incorporates McClelland’s Three Needs Theory and extends [27] organizational climate theory by introducing organizational climate as a moderator. The literature search employed key terms such as ‘Job Satisfaction,’ ‘Psychosocial Safety Climate,’ ‘Management Commitment,’ ‘Management Priority,’ ‘Organizational Communication,’ ‘Organizational Participation,’ and ‘Organizational Climate.’ Relevant studies were selected through searches on ‘Google Scholar,’ ‘Academia.edu,’ ‘Emerald Insight,’ and ‘Researchgate.’

This study is being conducted at Kota Kinabalu City Hall, Sabah, a municipal entity comprising a total population of 1842. A quantitative approach was employed using a questionnaire survey method to achieve the objectives of examining relationships among variables. The sample consists of 340 employees selected through a simple random sampling method across 19 departments, based on the [47] sampling formula. The selection process involved a computer-generated random number sequence, aligned with unique identifiers, to ensure an unbiased selection of participants, irrespective of department or role, minimizing systematic error. The sample size determination, following, uses a sampling error of 5% (significance level .05) with a confidence level of 95%. According to the calculations, for a total population not exceeding 1900, a minimum sample size of 320 respondents is required. The determination of the sample size is supported by the total target population of the study, based on the overall DBKK employee statistics provided by the DBKK Human Resource Development Department, which is 1842 people. Therefore, a sample of 340 respondents was used to further strengthen the study’s findings.

To initiate the questionnaire administration process, it was crucial for the researcher to establish rapport with the potential study participants. This was achieved by outlining the overarching aims of the research, explaining the significance and objectives of the study, and ensuring the confidentiality of the information provided by the respondents. A critical step in this preliminary phase was obtaining informed consent from the DBKK employees, thereby authorizing their participation as respondents in the study. Following their agreement, the questionnaires were personally distributed to the targeted respondents. The distribution and subsequent collection of these questionnaires were carried out over a three-week period, ensuring direct engagement with the participants and facilitating the data collection process.

The sample consisted of 52.6% females and 47.4% males, primarily aged between 31 and 40 (45.6%). The distribution across job roles was as follows: manual labor (4.4%), support II (71.2%), Support I (16.2%), managerial and professional (8.2%). Regarding the tenure in their current organization, 37.6% of respondents had served for less than 10 years, 28.2% for 11 to 20 years, 19.7% for 21 to 30 years, and 14.4% for more than 31 years. Almost half of the respondents had completed the Malaysian Higher School Certificate (SPM) or Malaysian Certificate of Education (MCE).

This study adopted the Standard Back-Translation (SBT) process [48] to ensure the content and face validity of all the scales. Following this process, the author engaged an associate professor in psychology to translate the original language of the questionnaire from English into Malay. Subsequently, the author invited a second translator (an English teacher) to translate it back from Malay to English. To ensure the equivalence of the translation, the author and the translators compared the interpretations until all errors were eliminated, and the questionnaire appeared reasonable and acceptable. A pilot test of the survey was then conducted with the participation of 25 employees at Kota Kinabalu City Hall, Sabah. Respondents in the pilot test re-evaluated the clarity of the translation, leading to some items being reworded, refined, or changed to be more understandable.

In this study, job satisfaction is measured using the ‘Job Satisfaction Survey’ (JSS) [49], employing a Likert five-point scale ranging from ‘completely disagree’ to ‘completely agree,’ with a Cronbach’s alpha of 0.782. Job satisfaction is treated as a single construct. The Psychological Safety Climate is assessed using ‘The Psychological Safety Climate Scale’ (PSC-12) developed by [5], utilizing a Likert five-point scale from ‘completely disagree’ to ‘completely agree.’ The scale includes four domains: management commitment, management priority, organizational participation, and organizational communication, each with Cronbach’s alpha values of 0.837, 0.780, 0.774, and 0.715, respectively. Organizational climate is measured with the ‘Organizational Climate Questionnaire’ (LSOCQ) developed by Nazar [26], employing a Likert five-point scale from ‘completely disagree’ to ‘completely agree,’ with a Cronbach’s alpha of .706. Organizational climate is considered as a single construct.

The collected data is analyzed using IBM SPSS Statistics and IBM SPSS AMOS. The analysis was indeed performed using SEM techniques, with the software AMOS been used for this purpose.

**Fig. 2:**
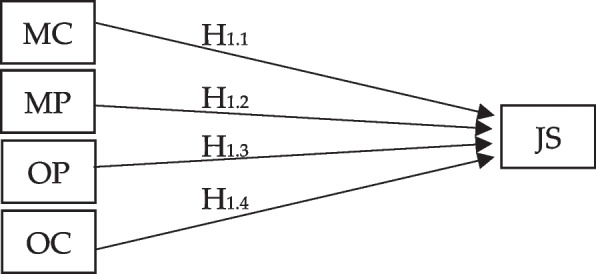
Linear regression analysis

**Fig. 3:**
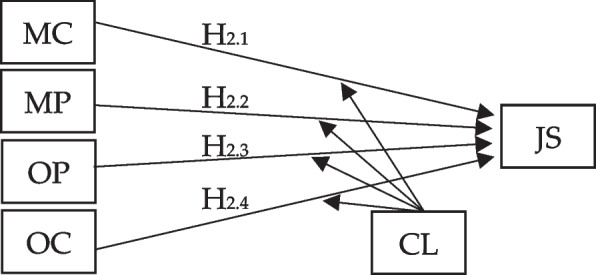
Structured equation modelling

Regression analysis is employed to examine the relationship between job satisfaction and each aspect of the Psychological Safety Climate (PSC), including management commitment, management priority, organizational participation, and organizational communication (refer to Fig. 2). Additionally, Structural Equation Modeling (SEM) is utilized to investigate the moderating effect of organizational climate on the relationship between each aspect of PSC and job satisfaction (refer to Fig. 3).

## Results and discussion

### Descriptive statistics

Table 1 present a total of 340 respondents consisting of Kota Kinabalu City Hall staff, Sabah were involved in this study. Table 4 shows that the respondents involved are 47.4% male, while the remaining 52.6% are female respondents. Respondents who are less than 30 years old have the smallest number of 13.8% followed by those aged 51 and over with 17.1% and respondents aged 41 to 50 years old with 23.5%. Respondents aged 31 to 40 years have the largest value of 45.6%. The respondents consisted of manual workers (4.4%), support II (71.2%), support I (16.2) and management and professionals (8.2%). More than one-third of respondents have served less than 10 years (37.6%), as well as respondents who have served 11 to 20 years (28.2%). While respondents who have served 21 to 30 years are 19.7% followed by respondents who have served more than 31 years by 14.4%. Almost half of the respondents have the highest level of education Sijil Pelajaran Malaysia (SPM) or Malaysian Certificate of Education (MCE). In addition, respondents are in the top 20% of income groups.

**Table 1:** Descriptive statistics results. Respondent background

Demographics	Frequency	Percentage (%)
Gender		
Men	161	47.4
Female	179	52.6
Total	340	100
Age group		
Less than 30 years	47	13.8
31 until 40 years	155	45.6
41 until 50 years	80	23.5
51 and above	58	17.1
Total	340	100
Service Category		
Manual workers	15	4.4
Support staff level II	242	71.2
Support staff level I	55	16.2
Management And Professional	28	8.2
Total	340	100
Length of service		
Less than 10 tahun	128	37.6
11 to 20 years	96	28.2
21 to 30 years	67	19.7
31 years and above	49	14.4
Total	340	100
Level of Education		
SRP/PMR	29	8.5
SPM/MCE	162	47.6
STP/STPM/HSC	29	8.5
Diploma	55	16.2
Bachelor Degree	54	15.9
Masters and above	11	3.2
Total	340	100
Income		
RM1,500 less	84	24.7
RM1501 to RM2500	100	29.4
RM2501 to RM3500	87	25.6
RM3501 and above	69	20.3
Total	340	100

*Source:* [50]

Table 2 presents the results of descriptive statistics aiming to assess the respondents’ agreement levels regarding job satisfaction, psychological safety climate dimensions, and organizational climate. Overall, this study reveals that respondents reported a moderate level of job satisfaction (Mean = 3.2653, SP = 0.30126). Additionally, respondents expressed a moderate perception of psychological safety climate dimensions, including management commitment (Mean = 3.4246, SP = 0.71492), management priorities (Mean = 3.5010, SP = 0.70254), and organizational participation (Mean = 3.3696, SP = 0.65258). Among the psychological safety climate aspects, organizational communication garnered the highest level of agreement (Mean = 3.6962, SP = 0.60486). Meanwhile, the organizational climate was assessed to be at a moderate level (Mean = 3.3027, SP = 0.31221).

**Table 2:** Descriptive statistics results

Variables	Mean	Std. Deviation	Level	Skewness	Kurtosis
Job Satisfaction	3.265	0.301	Moderate	0.423	− 0.167
Management Commitment	3.425	0.715	Moderate	−0.506	0.064
Management Priority	3.501	0.703	Moderate	−0.369	0.020
Organizational Participation	3.370	0.653	Moderate	−0.12	0.061
Organizational Communication	3.696	0.605	High	−0.267	0.036
Organizational Climate	3.303	0.312	Moderate	0.232	−0.187

*Source:* [51]

### Assumption testing

**Fig. 4:**
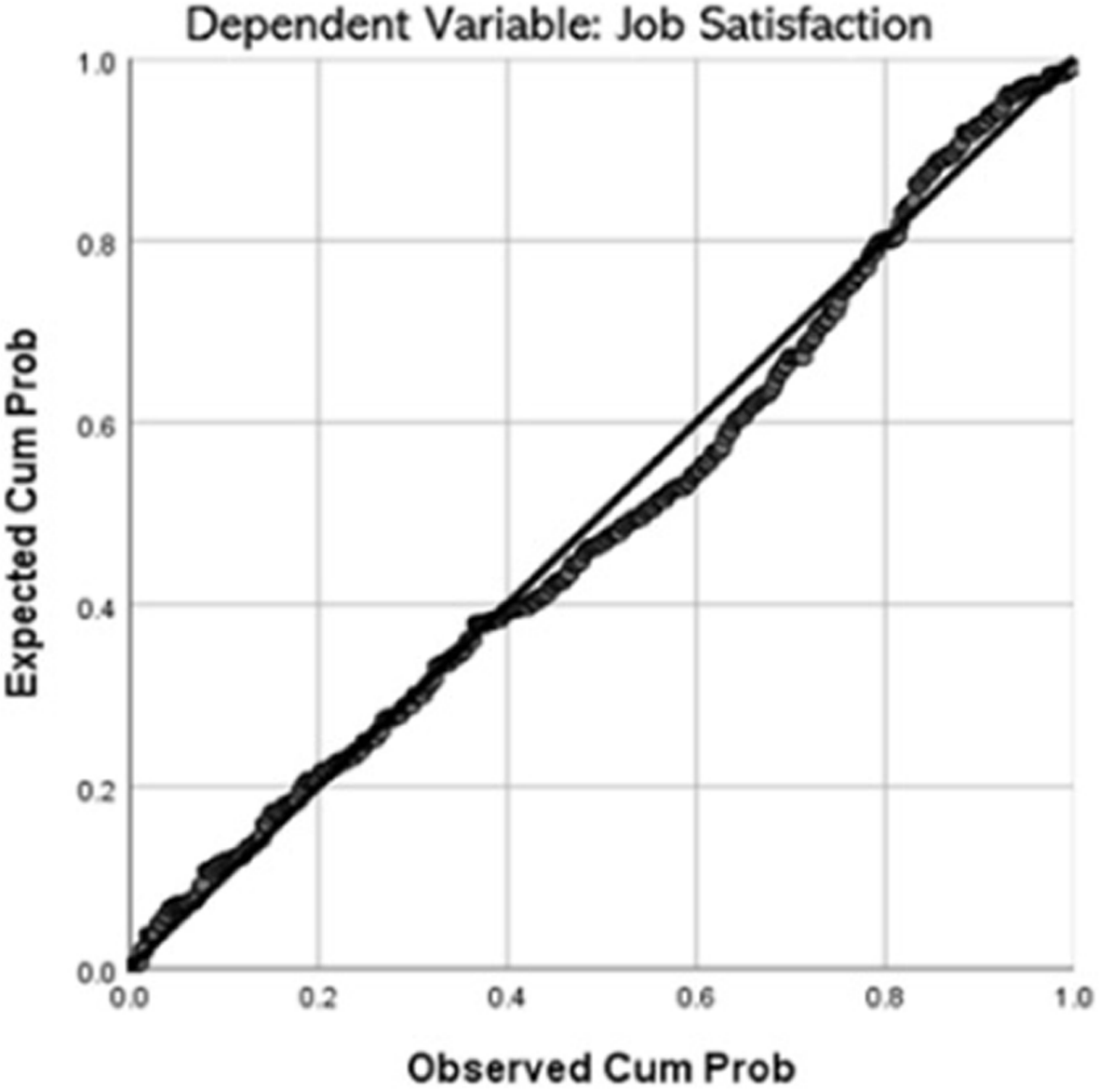
Normal P-P plot. *Source:* [51]

Referring to Table 3, the skewness and kurtosis values falling within the range of − 2 and 2 suggest a normal distribution of the data. Additionally, the Jarque-Bera test yields a non-significant value (F = 1.922, *P* = 0.3826) exceeding 0.05, reinforcing the conclusion of a normal data distribution. Figure 4 illustrates a scatter plot with points forming a straight line from the bottom left to the top right diagonally, indicating a positive linear relationship between the variables.

**Table 3:** Diagnostic test

Test	F-Statistics	Probability
Jarque-Bera	1.922	0.383
Breusch-Pagan-Godfrey	18.818	0.000

*Source*: [51]

**Fig. 5:**
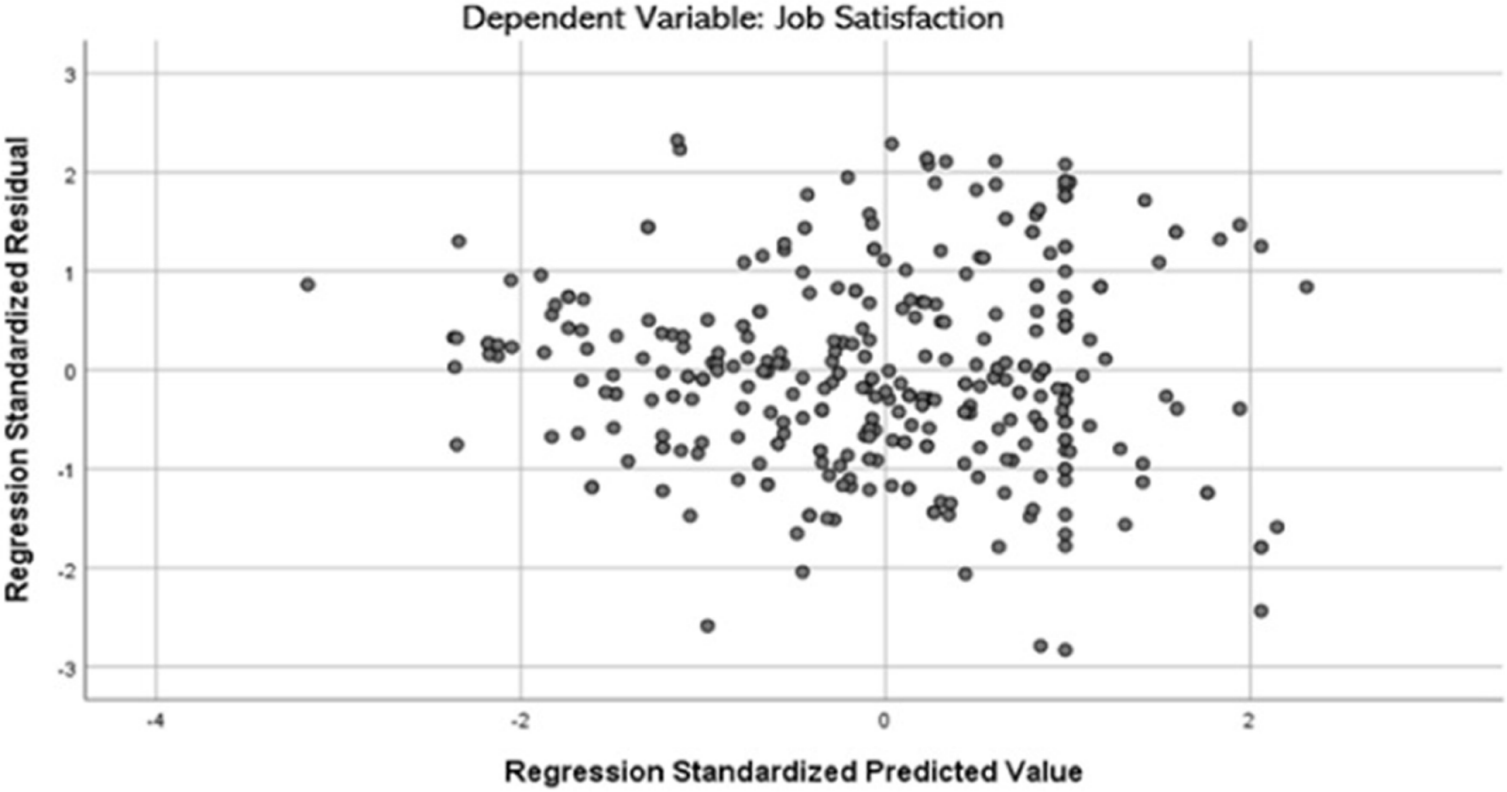
Scatterplot. *Source:* [51]

The VIF values in the models fall within the acceptable range (1 < VIF < 10), and the tolerance index (TI > 0.10) suggests the absence of a serious collinearity problem in the model (refer to Tables 3 and 4). However, based on the Breusch-Pagan-Godfrey Test, the *p*-value is less than 0.000 (Chi-Square = 18.818, *P* < 0.01), indicating that the regression model is heteroskedastic. The data distribution plots (Fig. 5) do not exhibit a spread and form a discernible pattern, confirming the presence of heteroskedasticity. To address this issue, the researcher employed the Wild Bootstrap approach for conducting linear regression analysis.

**Table 4:** Regression analysis coefficient

Variables	Job Satisfaction		Collinearity Statistics	
	Coefficient	*P*-Value	TI	VIF
Management Commitment (MC)	0.047	0.132	0.470	2.126
Management Priority (MP)	−0.010	0.773	0.369	2.709
Organizational Participation (OP)	0.151	0.001	0.397	2.517
Organizational Communication (OC)	−0.089	0.008	0.534	1.873

*Source:* [51]

Moreover, the study identified no outliers in the dependent variable (job satisfaction) and the independent variables (management priority and organizational participation). However, three outliers were observed in the independent variables: management commitment (1 Outlier), organizational communication (2 Outliers), and the moderator organizational climate (2 Outliers). No extreme outliers, exceeding 3 plot distances from the Boxplot boundary, were detected. Since no IDs are at extreme points, they are retained without any changes to maintain data integrity and preserve variation.

### Hypotheses testing

The results of the regression analysis in Table 4 reveal that management commitment (β = 0.047, *P* > 0.05) and management priority (*β* = − 0.010, *P* > 0.05) are statistically insignificant regarding job satisfaction. Consequently, H1.1 and H1.2 are rejected. On the other hand, organizational participation (β = 0.151, *P* < 0.01) has been demonstrated to positively contribute to job satisfaction. This implies that an increase in organizational participation leads to a corresponding increase in job satisfaction by 0.151, validating H1.3. However, organizational communication (β = − 0.089, *P* < 0.01) is statistically significant with job satisfaction but exerts a negative impact. In other words, an increase in current organizational communication by a unit is associated with a decrease in job satisfaction by 0.089.

When organizational climate is introduced into the relationship as a moderator through Structural Equation Modeling (SEM) to reinforce the role of psychological safety climate in enhancing job satisfaction, the moderating role serves to strengthen the relationship between management commitment (β = 0.106, *P* < 0.05) and organizational participation (β = 0.095, *P* < 0.05) with job satisfaction. The analysis in Table 5 reveals that the moderating effect of organizational climate positively influences management commitment toward job satisfaction. This implies that an increase in the interaction between organizational climate and management commitment by a unit will result in a job satisfaction increase of 0.047. Similarly, the moderating effect of organizational climate on organizational participation is proven to be positively significant with job satisfaction; a unit increase in this interaction will increase job satisfaction by 0.095. Consequently, H2.1 and H2.3 are validated. However, the moderating effect does not strengthen management priority (β = 0.008, *P* > 0.05) and organizational communication (*β* = − 0.015, *P* > 0.05), which are proven to be statistically insignificant at all levels with *p*-values exceeding 0.10%. Therefore, H2.2 and H2.4 are rejected.

**Table 5:** Moderated effect of organizational climate

Variables	Job Satisfaction		Collinearity Statistics	
	Coefficient	*P*-Value	TI	VIF
Organizational Climate * MC	0.106	0.019	0.979	1.021
Organizational Climate * MP	0.008	0.869	0.939	1.065
Organizational Climate * OP	0.095	0.040	0.940	1.064
Organizational Climate * OC	−0.015	0.789	0.959	1.042

*Source:* [51]

## Discussion

### The effect of psychosocial safety climate on job satisfaction

In the current relationship between management commitment, management priority and job satisfaction were statistically insignificant. Whereas organizational participation, organizational communication and job satisfaction where statistically significant.

In the current study, the relationships between management commitment and job satisfaction (β = 0.047, *P* > 0.05) was found to be statistically insignificant. This contrasts with findings from previous studies [33, 34], which established a positive relationship between management commitment and job satisfaction. The divergence in results may be attributed to specific challenges faced by Kota Kinabalu City Hall, such as an expanding service coverage area coupled with a decreasing redeployment rate, leading to employee fatigue. Despite efforts to increase management commitment, it did not significantly impact the job satisfaction of fatigued employees.

Similarly, the statistically insignificant relationship between management priority and job satisfaction (β = − 0.010, *P* > 0.05) contradicts previous research [35, 36], which asserted that effective management priority and leadership improve employee job satisfaction and organizational performance. The discrepancy could be linked to an increasing workload that surpasses the available workforce. Although Kota Kinabalu City Hall prioritizes the psychological health and safety of employees over productivity goals, an overwhelming workload may lead to fatigue and decreased motivation.

On the other hand, the study found a significant relationship between organizational participation and job satisfaction (β = 0.151, *P* < 0.01) with a positive coefficient value of 0.151. This aligns with research by [37] who observed a highly significant relationship between employee participation and job satisfaction among assistant managers and managers. Another study [38]. also supported this view, emphasizing the positive impact of organizational participation on job satisfaction and recommending that organizations prioritize employee and manager involvement for improved efficiency and performance.

Furthermore, a significant relationship between organizational communication and job satisfaction (β = − 0.089, *P* < 0.01) was observed, with a negative coefficient value of − 0.089. This aligns with studies by [39, 40], indicating a substantial connection between organizational communication and job satisfaction. However, the current study’s results diverge in the expected direction, suggesting that existing organizational communication practices may be poor and in need of revision.

### The moderating role of organizational climate on the association between psychosocial safety climate and job satisfaction

In the current study, organizational climate demonstrated statistically significant moderation between management commitment, organizational participation, and job satisfaction. However, organizational climate was found to be statistically insignificant in moderating the relationships between management priority, organizational communication, and job satisfaction.

The moderation role of organizational climate (β = 0.106, *P* < 0.05) indicates that the job satisfaction of Kota Kinabalu City Hall employees increases with the enhanced interaction between organizational climate and management commitment. This finding aligns with a study by [41], which reported that organizational climate significantly influences job satisfaction and organizational commitment, attributing 57.8% of the variance to organizational climate. Their research indicated that organizational climate positively affects work performance indirectly through organizational commitment [41, 42].

Similarly, the relationship between organizational communication and job satisfaction (β = 0.095, *P* < 0.05) suggests that the job satisfaction of Kota Kinabalu City Hall employees improves with increased interaction between organizational climate and organizational participation. This result is consistent with the findings of [43, 44], who identified organizational climate as a significant moderator between leadership influence and organizational participation.

Contrary to expectations, organizational climate did not exhibit statistically significant moderation between organizational communication and job satisfaction (β = − 0.015, *P* > 0.05). This finding contrasts with studies conducted by [45, 46], which reported simultaneous effects of organizational communication, organizational climate, and transformational leadership on employee job satisfaction. Additionally, [47–50] found that various aspects of organizational climate, including structure, communication, responsibility, appreciation, and reward, positively influenced employee productivity.

The moderation effect of organizational climate between management priority and job satisfaction, similar to organizational communication (β = 0.008, *P* > 0.05), contradicts the findings of [51, 52]. Their study suggested that organizational climate and organizational priorities collectively influenced employee work performance [53, 54], with an R Square value of 93.7%, indicating that only 6.3% of the variance was influenced by other variables.

Psychosocial safety climate is important among civil servants in Malaysia because it influences various aspects of their work experience, ranging from well-being and job satisfaction to productivity and organizational reputation [55]. Creating a positive psychosocial safety climate is an investment in the health and effectiveness of the public sector workforce. A positive psychosocial safety climate can contribute to higher employee retention rates. Civil servants are more likely to stay in an organization where they feel supported and valued. Additionally, it can enhance the organization’s attractiveness in recruiting new talent. Above that, a focus on psychosocial safety encompasses aspects such as managing workload, providing social support, and addressing issues related to work-life balance. These factors contribute to a healthier and safer workplace, reducing the risk of stress-related health issues among civil servants.

### Research implications

#### Theoretical implications

Our research significantly contributes to the theoretical landscape by delineating the intricate interplay between psychosocial safety climate and job satisfaction within the distinctive cultural and operational context of Malaysia’s public sector. The compelling evidence we’ve uncovered underscores the pivotal role of organizational participation in fostering job satisfaction, emphasizing the critical importance of employee engagement for cultivating a positive work environment. Simultaneously, our findings challenge conventional wisdom by revealing an inverse relationship between organizational communication and job satisfaction, suggesting that the effectiveness of communication strategies varies across cultural contexts.

Furthermore, this study boldly explores uncharted territory by delving into the moderating effect of organizational climate on the relationship between psychosocial safety climate and job satisfaction. The scarcity of empirical examinations in this domain, particularly in the Malaysian context, positions our research as a pioneering step towards unraveling these intricate dynamics. Our study not only expands the academic discourse but also provides practical insights for organizations seeking to optimize psychosocial safety climates and enhance job satisfaction within diverse cultural frameworks**.**

### Practical implications

The revelation of only moderate levels of job satisfaction among employees serves as a compelling call to action for organizations to reassess and fortify their strategies surrounding psychosocial safety. The relationship between organizational participation and job satisfaction is particularly illuminating, underscoring the significance of cultivating an environment where employees feel integrally involved and genuinely heard.

Moreover, the study’s insights regarding organizational communication reveal a paradoxical trend: instead of enhancing job satisfaction, current communication practices may be inadvertently eroding it. This divergence from expected outcomes suggests that there’s substantial room for improvement in the way communication is facilitated within organizations, and management must critically evaluate and overhaul these channels to create a more supportive environment for employee satisfaction.

The role of organizational climate as a powerful moderator also comes to the fore, particularly concerning the significant positive impact it has when combined with management commitment on job satisfaction. This finding encourages management to look beyond direct strategies and consider the nuanced ways in which organizational policies, employee autonomy, and a comprehensive rewards system can synergistically contribute to enhancing job satisfaction.

Additionally, the study briefly examines the less straightforward relationship between management’s prioritization of psychosocial safety and job satisfaction. The lack of a significant direct correlation could be indicative of broader organizational issues, such as understaffing, which could be causing widespread employee exhaustion. This highlights an urgent need for organizations to implement strategic talent acquisition and retention plans to ensure that the workforce is robust, satisfied, and well-equipped to handle their responsibilities.

## Limitations and future research

One limitation of this study is the use of a cross-sectional method for data collection, conducted at a single point in time. This approach may not capture the patterns of change and the full extent of causal relationships between the variables studied. The lack of control over variables can hinder the establishment of causal relationships, as the data collected at one time may not necessarily reflect the same results at another time. While common in research, the cross-sectional method limits the ability to make causal inferences and constrains the explanatory depth of the study’s findings. Therefore, future research could benefit from adopting a longitudinal study design, extending over a more extended period and utilizing the same sample at each phase to strengthen causal inferences.

Additionally, this study relies on a quantitative approach through a questionnaire survey. Future research should consider complementing this with a more in-depth qualitative approach to gain a richer understanding of respondents’ perspectives. The integration of both quantitative and qualitative methods can offer a more comprehensive explanation of the factors influencing employee job satisfaction in the public sector, along with insights into how organizational policies and practices impact these factors.

The study’s scope was limited to respondents from public sector organizations in Malaysia, specifically Kota Kinabalu City Hall. Replicating the research in different contexts is essential to validate reported differences and relationships. Future studies could apply the proposed conceptual framework to various organizations, including those in the private sector, non-governmental organizations, and statutory bodies.

Some non-significant relationships between independent variables and job satisfaction in this study could be further investigated by specifically measuring aspects of job satisfaction. However, concerns about survey length were taken into account in this study, as excessive questions may impact respondent cooperation and lead to careless responses.

Given the study’s context in Malaysia, where work culture emphasizes respect for leaders and organizations, there may be a potential for social desirability bias in respondents’ feedback. While efforts were made to mitigate this bias through anonymous surveys, future studies could expand the scope to other Asian countries and explore a cross-cultural perspective. National cultures differ, and a comparative analysis may provide valuable insights into cultural influences on job satisfaction perceptions.

## Conclusion

The study’s findings highlight that among the four aspects of psychological safety climate (management commitment, management priority, organizational participation, and organizational communication), organizational participation emerged as the most crucial variable, demonstrating a significant positive influence on job satisfaction. Conversely, organizational communication was identified as the second most important factor, exerting a significant negative impact on job satisfaction. In contrast, management commitment and management priority were found to be less influential, showing no significant effect on the job satisfaction of Kota Kinabalu City Hall employees.

Furthermore, when organizational climate was introduced into the relationship to reinforce the role of psychological safety climate in enhancing job satisfaction, the moderating effect strengthened the relationship between management commitment and organizational participation with job satisfaction. However, even with the moderating effect of organizational climate, management priority remained statistically insignificant. This indicates that organizational climate indirectly influences the effectiveness of psychological safety climate by impacting job satisfaction.

The literature review reveals mixed findings on the impact of organizational climate on psychological safety climate and job satisfaction, with limited studies conducted in Southeast Asia. These inconsistencies underscore the need for future researchers to contribute to the literature. Additionally, the researcher encourages future studies to expand the sample to include other public sector organizations to provide a more comprehensive understanding of the factors influencing job satisfaction among civil servants.

## Data Availability

According to need and permission.
